# Hide and seek with falsified medicines: Current challenges and physico-chemical and biological approaches for tracing the origin of trafficked products

**DOI:** 10.1016/j.forsciint.2025.112474

**Published:** 2025-04-15

**Authors:** Carla Perez-Mon, Cathrin Hauk, Alberto Roncone, Luana Bontempo, Simon D. Kelly, Céline Caillet, Michael Deats, Rob Ogden, Paul N. Newton

**Affiliations:** a Royal (Dick) School of Veterinary Studies and the Roslin Institute, University of Edinburgh, Midlothian EH25 9RG, United Kingdom; b NDM Centre for Global Health Research, Centre for Tropical Medicine and Global Health, Nuffield Department of Medicine, University of Oxford, Oxford, United Kingdom; c Medicine Quality Research Group, Centre for Tropical Medicine and Global Health, Nuffield Department of Medicine, University of Oxford, Oxford, United Kingdom; d Infectious Diseases Data Observatory, Centre for Tropical Medicine and Global Health, Nuffield Department of Medicine, University of Oxford, Oxford, United Kingdom; e Mahidol Oxford Tropical Medicine Research Unit, Faculty of Tropical Medicine, Mahidol University, Bangkok, Thailand; f Traceability Unit, Research and Innovation Centre, Fondazione Edmund Mach, Via E. Mach 1, San Michele All’Adige, TN 38098, Italy; g Food Safety & Control Laboratory, Joint FAO/IAEA Centre of Nuclear Techniques in Food and Agriculture, International Atomic Energy Agency, Vienna International Centre, Wagramer Strasse 5, P.O. Box 100, Vienna 1400, Austria; h TRACE Wildlife Forensics Network, Edinburgh EH12 6LE, United Kingdom

**Keywords:** Falsified medicines, Route tracing, Geolocation, Forensics, Spectrometry, EDNA

## Abstract

The criminal trafficking of falsified medical products is a worldwide, yet still largely overlooked, public health problem. A falsified medicine fraudulently misrepresents its identity, composition and/or source, often being ineffective or toxic for patients. Although techniques have been developed to detect falsified medicines, it remains a challenge to trace where- and by whom- the products are manufactured. We aim to discuss plausible biological and physico-chemical analytical techniques that could reveal information about the origin of medical falsifications. We first provide a brief overview on the prevalence, criminal activities, health impacts and (bio) chemical features of falsified medical products. We then explore diverse laboratory approaches, that are used in food fraud, illicit drug and wildlife trafficking investigations, and discuss how they could be combined and redirected towards tracing falsified medicine origin and hence empowering enforcement to counter this pernicious but neglected global health problem.

## 1 Introduction

### 1.1 Definition and insights into the history, trade and societal impacts of falsified medicines

The trade in falsified medical products, here focusing on medicines and vaccines, is one of the largest and probably most lucrative fraud markets in our globalised world, and is a major but neglected impediment to the achievement of the Sustainable Development Goal 3 of the United Nations: *equitable access to safe, effective, quality and affordable essential medicines and vaccines (target 3*.*8)* [1,2]. According to the definition of World Health Organization (WHO) [3–5], a falsified medicine (FM) is a “poor quality” product which fraudulently misrepresents its identity, composition or source. The World Health Assembly agreed to use the term falsified to highlight the public health importance of such fraud, rather than the term counterfeit that focuses on intellectual property rights [4]. Falsified medical products should not be confused with substandard medical products, which are authorized products of poor quality because of unintentional errors during manufacturing (e.g., inappropriate formulation) transport or storage [5].

In 2017 WHO estimated that 10.5 % of medical products in low- and middle-income countries (LMICs) are falsified or substandard [3]. In comparison, a meta-analysis conducted by Ozawa et al. [6], which included 265 studies, suggested that 13.6 % of medicines in LMIC are falsified or substandard, with a regional estimated prevalence of 18.7 % in African countries and 13.7 % in Asian countries. An even higher percentage of 19–50 % substandard and FMs has been estimated for the Sahel countries [7]. The epidemiological data are of poor quality and quantity, and there has been confusion between falsified and sub-standard, making it difficult to understand their comparative epidemiology [8,9].

The detrimental health and socioeconomic impacts of poor-quality medical products are vast and multidimensional. In sub-Saharan Africa up to 116,000 deaths per year have been estimated to be due to falsified or substandard antimalarials, and up to 169,000 deaths to poor quality antibiotics used to treat severe pneumonia in children worldwide [3]. Patients who consume these products may recover more slowly or not at all from treatable diseases, and, in the case of falsified antimi-crobials, the products may contribute to the development and spread of antimicrobial resistances [3,10]. The prevalence of poor-quality medicines not only contributes to higher rates of mortality and morbidity in affected populations, but they also exacerbate mistrust in public health systems and lead to economic losses [3].

Medicine falsifications are by no means a new phenomenon. Early reports of medicine falsifications describe the prevalence of falsified medical herbs in ancient Egypt bazaars (ca. 1500 BC; Kreig [11]), and adulterations (e.g., using debris and gypsum) of herbal medicines such as saffron in ancient Greece (ca. 65 AD; Dioscoridis’ Materia Medica, Kreig [11], Rooney [12], Chen et al. [13]). Following the end of World War II penicillin shortages led to falsified penicillin proliferation in black markets [14]. More recent examples described in the scientific literature are collated in the Medicine Quality Scientific Literature Surveyor (https://www.iddo.org/mq-scientific-literature-surveyor), such as the reporting of falsified COVID-19 vaccines in at least 36 different countries (Kerlijn Van Assche, personal communication, https://www.iddo.org/mq/research/medical-product-quality-reports).

Currently, the value of the trade in falsified medicines is estimated at US $70 to $200 billion annually [15]. High profits, a very low risk of detection or prosecution, and penalties much lower than compared to illegal drug trafficking may have made the trade in falsified medicines more attractive to criminals than trafficking in illegal drugs [16]. Traffickers range from small and larger scale criminal networks to health agency workers, or licensed pharmaceutical companies that produce legitimate medical products by day and falsified ones at night [17,18,16, 19,20]. Locally, the trade in FMs is promoted through a combination of high consumer demand for treatments that in many countries are inaccessible due to supply issues and/or cost, poor governance and weak technical medicine regulatory capacity [21]. At a global scale, the trade is facilitated by porous borders and a lack of transnational control mechanisms and collaborations among medicine regulatory, law enforcement agencies and pharmaceutical industries [4].

Furthermore, legal pharmaceutical supply chains are highly complex, often opaque and especially fragmented in LMICs, with multiple parallel distribution systems and a lack of international and national coordination [22]. This hampers end-to-end tracing of routes and creates opportunities for manufacturers to infiltrate and launder their FMs into the legal trade. An UNODC report indicated that between 2013 and 2021 around 40 % of the medical products in the regulated supply chain in the Sahel countries were estimated to be falsified or substandard [7], and it revealed high interconnectivity between the regulated and unregulated supply chains [22], [23], [24]. In high- and middle-income countries, the growing popularity of e-commerce and the practice of parallel trade in the EU, which allows for local re-packaging, represent potential entry points for falsifications [17,25].

### 1.2 Features of falsified medicines and their manufacturing

**Fig. 1:**
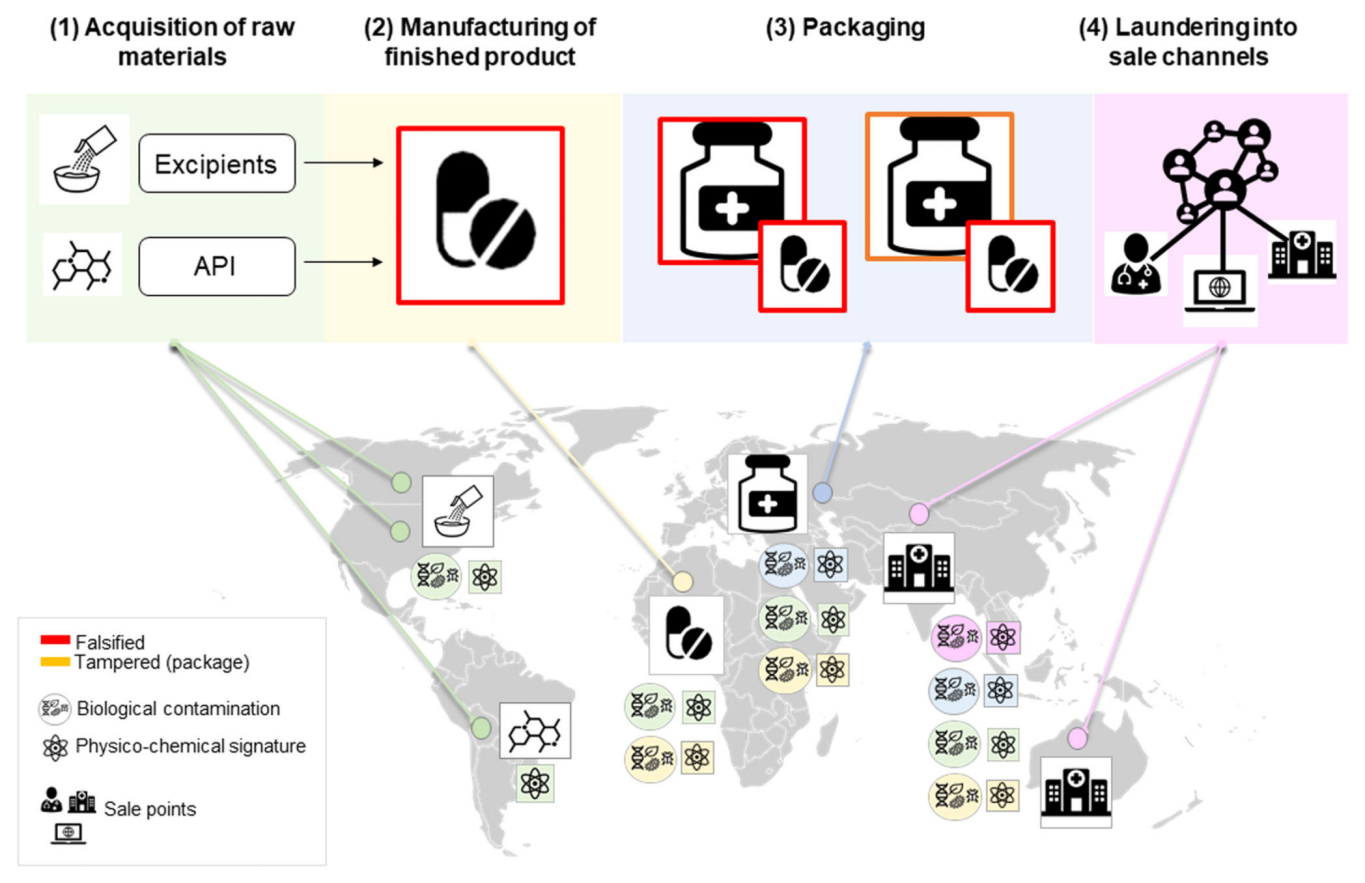
Schematic representation of the manufacturing process of falsified medicines and the provenance of their chemical and biological signatures. The different background colours represent distinct geographical locations, which in this illustration also correspond to separate manufacturing steps. Each manufacturing step potentially adds new chemical and biological signatures to the falsified medicines, shown by the sequential addition of differently coloured signatures. Locations depicted are for illustrative purposes only.

Manufacturing and distribution of FMs comprise some or all of the following four steps, that may not occur in this order or in the same place: (1) acquisition and transport of excipients and other raw materials to the manufacturing site, (2) manufacturing the finished product (3) manufacturing the packaging and (4) laundering into physical or virtual sales channels (Fig. 1). The “laundering” step can occur before the product is manufactured, e.g., in cases when the falsification is pre-ordered by traders with criminal ties [19]. FMs are highly heterogenous, and falsifications may concern the medicine itself (tablet, capsule, vial content etc.) and/or the packaging and labelling [26,27,19]. Based on the composition of the finished product, falsifications can be divided into those that: (a) do not contain an active pharmaceutical ingredient (API), (b) contain the wrong amount (reduced or increased) of the stated API, or (c) contain API that are not declared on the packaging [28–30], unexpected excipients such as flour, calcite and talc [30] or (toxic) impurities [27].

Packaging of the finished product frequently involves the use of falsified packages. Packaging falsification has become easier in the last decades due to high quality printing and packaging technology becoming more affordable and accessible to criminals [19]. Re-used genuine packaging has also however been observed in medicine falsifications. Common practices include reusing or modifying (i.e., tampering) genuine containers, e.g., by extending the expiry date [31, 26,29].

### 1.3 Where do falsified medicines come from?

Numerous detection techniques are employed to detect and intercept FMs in the supply chain, as listed in Table 1 and reviewed in Fernandez et al. [32], Vickers et al. [33], Lanzarotta et al. [34]. The outstanding challenge and the focus of this review is that: once falsification is identified, e.g. through seizures or surveys: *how to trace back where, and by whom were they produced?* Traditional criminal investigations have been the mainstay and some of these, especially those with successful prosecution, are reported in the press. However, there is little objective epidemiological evidence in the public domain as to the geographical origin of falsified medicines. Adaptation of techniques used in food fraud and the illegal narcotics and wildlife trades offer promise for enhancing such investigations.

Genuine finished pharmaceutical products usually represent a composite of diverse geographical origins (e.g., APIs mostly come from India and China; and excipients from Europe and North America [52,53]). To complicate matters, manufacturing and packaging of medical products often take place in yet another country. Similarly, the raw materials of a FM can originate from very different parts of the world (Fig. 1). There is clearly great variation in types of ‘facilities’ in which falsified medicines are ‘manufactured’ from lone criminals’ kitchens to more sophisticated interlinked factory networks.

The trading networks of falsified medical products, both between illegal traffickers and/or between legitimate traders unaware of falsifications, are often multinational and convoluted, which aggravates the “delocalization”, or lack of understanding of the diverse origins of the falsified goods. A notable example is a FM labelled as Avastin® (bevacizumab) which reached the USA via dubious trade routes across three continents and was uncovered because it showed no benefit in cancer patients [4].

### 1.4 What can we do to localize falsified medicines? Aims of this review

The “delocalization” of falsified medical products is a major impediment to their eradication. Their forensic investigations require robust collaborations between law enforcement and academic entities, national medicine regulatory authorities, and, crucially, innovative and generic pharmaceutical companies [54,55,29]. Collaborative forensic analyses of FMs (and their packaging) can yield information, which, aggregated in multiple lines of evidence [56], could help addressing crucial questions, such as: Q1: *Does FM (A) (i.e., an unknown single sample or batch of samples) share its origin with another FM(B)?* Q2: *Where do the ingredients of FM(A) come from?* Q3: *Where was the FM(A) produced?*

Here we provide an overview of laboratory techniques that may expand our ability to answer these kinds of questions. We particularly focus on three innovative techniques which provide detailed information of physico-chemical and biological features of finished products and ingredients. Using case-work investigations on illicit drugs, food fraud, wildlife trafficking, and FMs pilot studies, we describe how such three streams of evidence could each help to uncover the geographic origin and supply routes of falsified pharmaceuticals. We also propose strategies to integrate the different forensic evidence for a more effective tracing of falsified medical products.

## 2 Analytical laboratory techniques to potentially trace falsified medical products

**Fig. 2:**
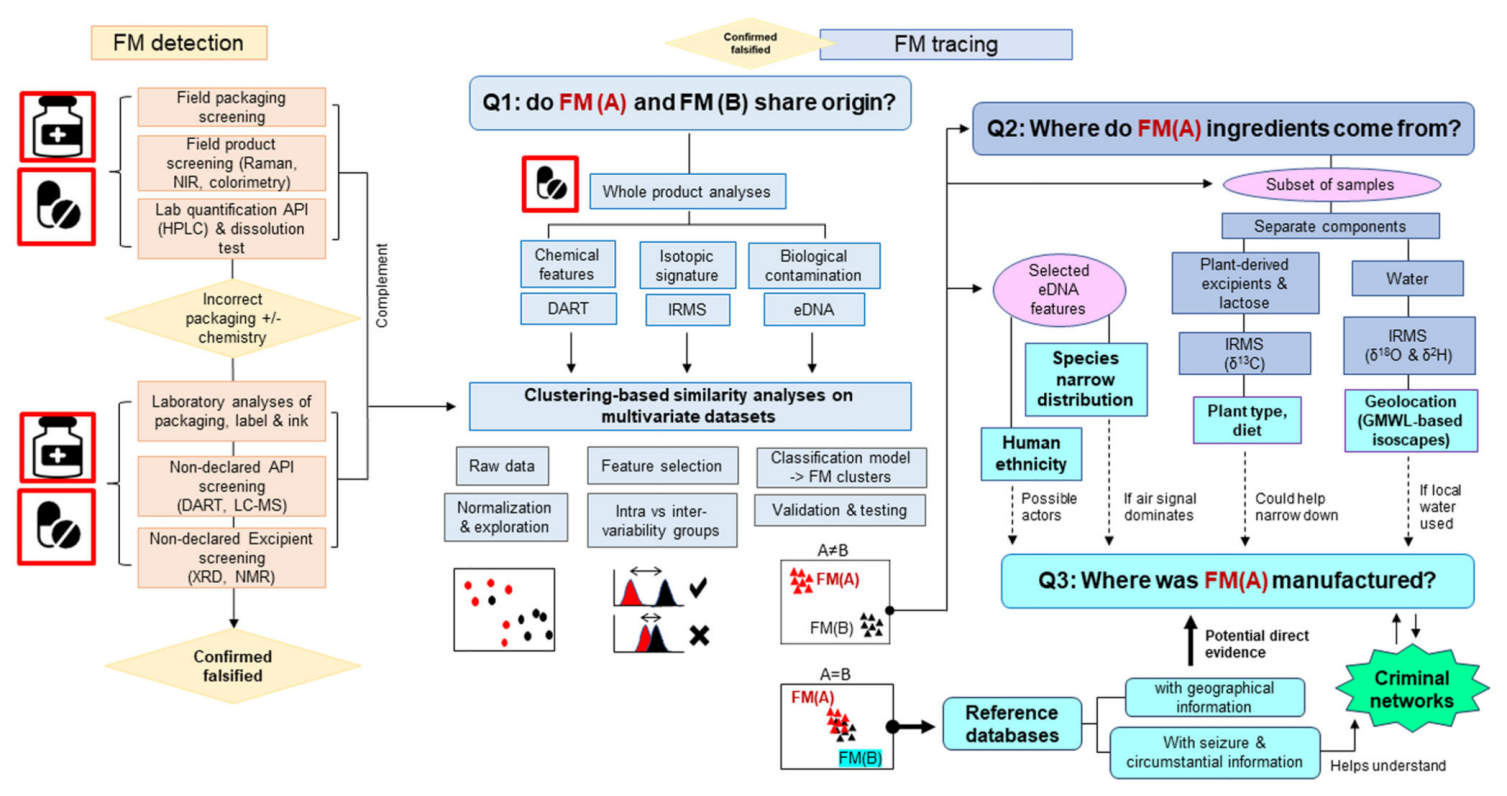
Workflow for the detection of falsified medicines and proposed tests and analytical strategies to geolocate the products. Based on Fernandez et al. [32], Dégardin et al. [54], Hochholdinger et al. [56]. FM: Falsified Medicine, Raman: Raman spectroscopy, NIR: Near-Infrared spectroscopy, API: Active Pharmaceutical Ingredient, HPLC: High-Performance Liquid Chromatography, DART: Direct Analysis in Real Time, LC-MS: Liquid Chromatography–Mass Spectrometry, XRD: X-ray diffraction, NMR: nuclear magnetic resonance spectroscopy, IRMS: Isotope Ratio Mass Spectrometry, eDNA: environmental DNA.

The first step when intercepting a suspected falsified medicine (FM) is to accurately establish that it is indeed falsified (Fig. 2). The primary and secondary packaging (e.g., blister pack and outer carton) of the products are visually inspected in search of spelling mistakes, incongruencies or graphical (i.e., logo, seals, colours) differences, both overt and covert, with respect to its genuine counterpart, if these are available [32,35]. Depending on the setting and the available resources, (portable) screening technologies and/or pharmacopeial analysis are applied. Screening techniques, e.g., colorimetry, Thin Layer Chromatography (TLC), Raman and Near-Infrared (NIR) spectroscopy are used in the field and in low-resource settings [33]. Pharmacopeial reference analysis, such as assay (content analysis) and dissolution testing are conducted to check whether the product meets the required specifications (e.g., HPLC identification and quantification of API(s)). These tests provide an initial layer of information that could be of potential use to establish linkages between FMs or with trade networks [57].

Once identified as falsified, they can be further explored through comprehensive laboratory analysis of its features and subsequent comparative data analysis to assign the product to clusters of common origin (of components or putative manufacturing facilities). Where possible it could indicate signatures of geographic localisation along its production and distribution pipeline (Fig. 2). High-resolution optical analysis of packing and printing using different light wavelengths and magnification (e.g., US FDA CD3 + device [58], FTIR (Fourier-transform infrared spectroscopy), VSC (Video Spectral Comparator) and Raman spectroscopy, can provide information about the packaging material and processing. Analyses of tablet dimension and surface (e.g., using calipers, profilometry and 2D and 3D tablet surface analysis such as scanning electron microscopy; SEM) might provide clues on the tablet press dies used; whereas analysis of its composition (e.g., chemical profiling with high-resolution spectrometry) provide details about their production [34,56]. Table 1 gives an overview of potential techniques that can be used for analysis of medical products and packaging, and their potential application(s) for tracing inter-sample relationships and origins.

In the following sections we will discuss three of the most innovative techniques to characterize FMs and inform relationships between samples and their origins: (1) Direct Analysis in Real Time (DART) MS as a non-destructive method to rapidly obtain in-detail chemical profiles of the FMs, (2) stable isotopic measurements as a means not only to potentially discriminate between products, but also to identify the origin of their components (i.e. excipients and API), and (3) environmental DNA (eDNA) metabarcoding as a fast, high-throughput method to characterize biological contaminants.

### 2.1 DART mass spectrometry

DART-MS is an ionization spectrometric technique that subjects a test sample under investigation to a stream of super-heated (300–500 °C) metastable gaseous atoms (typically helium or nitrogen). The heated stream desorbs chemical species from the sample and simultaneously ionizes them; through a series of ionizing reactions between the heated gas, atmospheric air (i.e., water and gases) and the sample. The resultant analyte ions are then transferred to the mass spectrometer by a pressure gradient and/or electric field, and separated

by their mass to charge ratio (^M^/_Z_) [42,59]. DART enables the analysis of a wide range of samples, i.e., food, drinks, drugs, biological tissues and explosives [40,60], [42], [61]. DART has the advantage, unlike other mass spectrometry techniques, that samples can be analysed in the open air, thereby requiring minimal preparation. Products can be placed in their native form directly into the beam, which largely preserves their integrity while speeding up analyses. As the technique results in minimal physical damage to the surface of the sample, limited quantities of material, as it is often the case for FMs, can be used for other follow-up analyses [40], [42].

DART ionization coupled with time-of-flight mass spectrometry (TOF-MS) is the preferred configuration for product authentication applications [42], as DART-TOF-MS can quickly produce high-resolution mass spectra for multiple compounds, including those contained in FMs [41,40,32]. Bernier et al. [41] screened 192 falsified antimalarial tablets collected in sub-Saharan Africa using DART-TOF-MS. The authors demonstrated the suitability of this technique to rapidly detect FMs while providing forensic insights into manufacturing patterns.

Multivariate analysis of the FM spectra together with compound identification distinguished two distinct classes of tablets: (1) those containing saccharides and (2) those containing chloramphenicol or ciprofloxacin; instead of the stated artemether-lumefantrine active ingredients. These two classes could indicate different origins in space and/or time of the studied FMs. Furthermore, some tablets from class (2) contained mixes of chloramphenicol and ciprofloxacin in varying proportions, suggesting manufacturers altered their formulation over time.

The application of DART data to falsified medical product sample origins can potentially be achieved through comparative analysis with other products or geographic source, where reference samples with provenance information are available. For instance, in wildlife forensics, Coals et al. [60] demonstrated the use of DART to distinguish between different sources of lion bone, including captive (legally traded) lions and protected wild individuals. DART profiles enabled discrimination of the two bone sources, suggesting the basis for an identification tool. It also led to the tentative identification of chemical compounds that are used in pharmaceuticals, such as imidazole, pyridine, and triazole derivatives, associated with the captive bred group.

Current limitations of DART-TOF-MS for FM product forensics centre on the inter-laboratory transfer of reference data and, at a more detailed level, the challenges associated with DART compound identification. The latter is frequently ambiguous and, in the absence of relevant standards for mass spectral confirmation, biased towards compounds already available in reference databases. For more robust identification of compounds, DART can however be complemented with other techniques such as gas chromatography (GC) and Liquid-chromatography-mass spectrometry (LC-MS) [62].

### 2.2 Stable isotope ratio analyses

Stable isotope analytical techniques rely on the natural occurrence of distinct isotopes of an element (e.g., O, H and C), at varying relative abundances (i.e., expressed as ratios; ^18^O/^16^O, ^2^H/^1^H, ^13^C/^12^C on the relevant delta (*δ*) scale calibrated with internationally accepted reference materials). Variations in isotopic ratios are influenced by climatic, geological, ecological and physiological factors [63,64]. As such, materials carry distinctive isotopic signatures which can inform about the environment in which they originated, the nature of their chemical structures and synthesis, or, for biological specimens, about life history and metabolic processes [64]. For instance, *δ^18^O* (^18^O/^16^O) and *δ^2^H* (^2^H/^1^H) isotopic ratios measured in the water contained in an investigated material can inform about its geographical origin, because *δ^18^O* and *δ^2^H* correlate to world locations (i.e., Isoscapes) through the Global Meteoric Water Line (GMWL) [65]. Additionally, δ^13^C can provide information on the botanical origin of plant-derived materials, such as common pharmaceutical excipients like cellulose or starch. This is because plants usually exhibit one of two main types of photosynthetic pathways (C3 vs C4 plants) through which fixed C fractionates differently, resulting in two non-overlapping ranges of *δ^13^C* values (− 22 ‰ to − 33 ‰ for C3 vs − 8 ‰ to − 16 ‰ for C4) [46].

IRMS (Isotope ratio mass spectrometry) is the principal technique used to resolve isotopic compositions, essentially consisting of an inlet device that converts a sample into a gas for measurement (typically H_2_, N_2_, CO, CO_2_, or SO_2_). The gases are passed into an IRMS where they are ionised and separated by an electro-magnetic sector according to their ^M^/_Z_ ratio, prior to quantification with dedicated Faraday collectors for very precise measurement of the isotopologue ratios [66]. Stable isotope analyses using IRMS have been commonly used in the forensics investigations of food fraud [67] and the illegal wildlife trade [68]. IRMS has also been used on a few occasions in tracking and authentication of pharmaceutical products [69,70,30], whereas recent studies go one step further, underlining the forensic potential of this technique to draw linkages between FMs based on multi-isotopic profiles and raw ingredient sources [46].

The stable isotope ratios of FMs are influenced by the natural isotopic variation of their raw materials (e.g., excipients, API and water), and isotopic fractionation that may occur during the manufacturing process. This means that it is virtually impossible to randomly recreate the multi-isotopic profile of a medicine (and intentionally doing so would cost more than legally producing the medicine) [71]. Hence, a primary approach in sourcing the provenance of a FM sample using IRMS is to measure the isotopic ratios of the medicine as it is, the main advantage being the avoidance of extensive sample preparation, significantly reducing analysis time. This approach allows for manufacturer-based discrimination among FMs of unknown origin, e.g., based on clustering of the combined C, H, O (and potentially N and S) stable isotope signatures [46,70]. This method could determine if suspicious material seized at different times or places originates from the same batch or manufacturer, for which geolocation information might or not exist.

A second approach involves the compound specific analysis of separated FM components [70,72]. This approach is more labour intensive, requiring the extraction and purification of the components, whose identity is often unknown, before IRMS measurements. It does however offer the advantage of providing more specific information on the raw materials’ provenance and, indirectly, the geographic origin of the product. For instance, falsified medicines have been found to exhibit higher δ^13^C values compared to genuine ones [70], which could be related to the use of excipients derived from C4 crops (e.g., starch derived from maize) that are more extensively grown in tropical and subtropical ecosystems [73]. Comparably, δ^13^C of the excipient lactose, which is used in pharmaceuticals, could inform about the type of diet (C3 or C4 based) of the animal from which they derive, which in turn could differ geographically [72]. Manufacturing of FMs can also include the use of local water (e.g., in tablets produced using the wet granulation method or falsified vaccines containing water), whose δ^18^O and δ^2^H values could point to the production site, assuming the water remains unfractionated by any short- or long-term storage conditions. Similarly, such analysis of water in falsified vaccines and diluents could provide actionable evidence of origin.

An alternative method to using IRMS is Nuclear Magnetic Resonance (NMR), widely used in abuse-drug forensics to gain insights into the synthesis origin of a product’s components [74,75]. In the case of APIs, different synthetic pathways yield molecules with distinct C and H isotopic ratios at specific positions. q^2^H NMR and ^13^C NMR can detect these position-specific variations, potentially allowing for batch differentiation among chemically identical pharmaceutical products [47,76]. For example, ^13^C NMR can effectively identify the synthetic routes of APIs and their precursors’ origins [47,77]. Isotopic values of ^13^C in central molecular regions, such as aromatic rings, can provide indications about the origin of the precursors (e.g., natural or petrochemical). In contrast, C atoms in side chains, which are strongly influenced by chemical reactions, reveal insights into the synthetic pathway of the API.

Certain limitations have to be considered for the applicability of stable isotope analyses in the context of FMs’ tracing. Firstly, isotope analyses should be complemented with other screening techniques to preselect samples for analyses and to aid interpretation of results. Secondly, a stage of method optimization is needed, ideally using samples of known composition and origin, to ensure the accuracy and precision of measurements and the statistical power of the technique to detect differences between samples’ batches. Lastly, when it comes to FM’s origin assignment, isotope analyses would ultimately rely on building robust global-scale databases with a suitable representation of real-world samples, with an understanding of any fractionation effects that could occur during manufacture, storage and degradation of samples.

### 2.3 eDNA metabarcoding

Non-human biological trace evidence; which includes evidence derived from plants, fungi, animals, protists or prokaryotes (i.e., Archaea, Bacteria and viruses), is a valuable source of information in forensic investigations [50], [78], [79]. Numerous plants and animal species exhibit relatively narrow geographic distribution ranges, or they are only present at high biomass at certain period of the year (e.g., annual plants). Hence, observations of plant and animal remains from crime scene materials can help identifying the habitat, geographical location, or even time at which a criminal activity took place [50], [78]. Microorganisms (e.g., prokaryotes and fungi) are generally widespread and adapted to multiple environments. Yet identification of microbial strains can help linking objects and actors to crime scenes [79,80], or to locate sources of contamination [81–83].

Biological trace evidence found in illegally manufactured products, such as FMs, is represented by a mixed community of eukaryotic and bacterial taxa; accrued through the -often unclean- manufacturing facilities, shed by the manufacturers, or contained in the equipment and materials used during fabrication [84]. Identification of biological taxa in forensic materials, including FMs [30], have been achieved through approaches such as palynological analyses and microbial culturing [50, 79]. However, these are labour-intensive techniques and taxonomy experts are scarce. The refinement of molecular DNA techniques together with the development of next-generation sequencing (NGS) technologies and databases during the last two decades has brought a new alternative to the forensics field: using environmental DNA (eDNA) to obtain biological profiles in a faster, and less expensive and sample-destructive manner [48].

eDNA refers to cell-free DNA, released into the “environment” by the damaged cells of organisms [85]. Modern eDNA extraction kits, based on DNA-binding to silica columns (e.g., QIAamp DNA Investigator Kit; Qiagen, Thermofisher) or magnetic beads (e.g., PrepFiler™ Forensic DNA Extraction Kit; DNA IQ™, Promega) currently enable the recovery of forensic eDNA from minimal amounts of sample (<0.1 g) and even if present in ng quantities, as in FMs. eDNA-based community species identification relies on linking the forensic eDNA to its original biological hosts through NGS and interrogation of the sequences against genomic-taxonomic databases. To this aim, there are two major approaches: metabarcoding and metagenomics.

Metabarcoding is based on the sequencing of specific regions in the DNA (a.k.a taxonomic markers) that are highly similar between individuals of a same species, but differ between different species. Meta-barcoding workflows involve the amplification of taxonomic markers via PCR, followed by the sequencing of the amplicons. Metagenomics consist of the sequencing of all gene content extracted from a sample, followed by a later *in-silico* reconstruction of large genomic regions, which would harbour multiple taxonomic markers for species identification. Sequencing is achieved using NGS platforms such as those provided by Illumina (miSeq, HiSeq, NextSeq, NovaSeq), Oxford Nanopore (MinIon, GridIon, PromethIon) and PacBio (Onso and Revio systems) (see Satam et al. [86] for detailed explanations of NGS platforms and their applications). The revolutionary power of NGS platforms, also referred to as “high-throughput” or “massive parallel”, resides in their capability to sequence multiple DNA samples in a single run, producing millions of reads.

Metabarcoding is arguably the most commonly used approach for DNA-based community species identification [87]. Common taxonomic markers for species identification include: the 16S ribosomal RNA gene for bacteria, the 18S ribosomal RNA gene for fungi, animals and protists, the mitochondrial cytochrome oxidase I (mtCOI) gene for animals, and the Internal transcribed spacer (ITS), between the small-subunit and large-subunit rRNA genes, for the identification of fungi and plants [88]. Plant-specific marker genes, present in chloroplasts, also include the trnL (UAA) intron, MaturaseK gene (MatK), the large subunit of the ribulose bisphosphate carboxylase (rbcl) gene, and the trnH–psbA intergenic spacer region [88]. Corresponding taxonomic databases have been built for these marker genes such as the 16S Greengenes database for bacteria (https://greengenes2.ucsd.edu/), 16S/18S-SSU SILVA database for bacteria and eukaryotes (https://www.arb-silva.de/), the 18S PR2 database for protists, fungi, plants and animals (https://pr2-database.org/) and the ITS Unite database for fungi (https://unite.ut.ee/). Other initiatives such as BOLD systems (https://www.boldsystems.org/) comprise curated mtCOI, ITS, rbcl and Matk for the identification of animals, fungi, and plants, whereas all-gene repositories such as NCBI enable to query any taxonomic marker.

Metabarcoding techniques have been applied only relatively recently in forensics. Pilot and feasibility studies conducted on falsified medicines, illicit drugs, herbal and food products have yielded promising results [89,90,84,49]. For instance, in a study conducted by Young et al. [49] on falsified antimalarials, the authors demonstrated the recovery of bacterial (16S rRNA marker), fungal, animal and plant (18S rRNA marker) DNA, which they were able to annotate to taxa. On one hand, the authors showed the capability to discriminate between genuine and falsified samples, and, more importantly, between different types of falsification, solely based on the direct comparison of their DNA profiles. This observation opens the possibility to detect linkages between seizures or even geolocate new batches of falsifications based on their similarity to previous samples that had geographical information. On the other hand, the authors detected species such as the fungi *Hortaea werneckii, Phellinus noxius, Flammulina velutipes*, which are either present or cultivated in the east Asian regions, as well as grasses such as *Zea mays* (corn) and *Triticum aestivum* (wheat), which are commonly used to produce excipients for medical products (e.g., starch). Hence, this points to the possibility of using the taxonomic identities to narrow down the possible distribution ranges or environments in which a product is manufactured. A similar rationale has been followed e.g., in honey origin authentication investigations, in which clustering-base methods applied to bacterial, fungal and plant sequences successfully discriminated between honeys produced in distinct neighbouring Scandinavian countries [23], [91], and DNA-based identification of plants helped in testing the stated regional provenance of honeys from Iran [92].

If global-wide bacterial genomic databases can be expanded these offer hope for metabarcoding of bacteria isolated from contaminated falsified medicines (and in this case also substandard products due to bacterial contamination within factory), to yield information on likely provenance for bacteria whose whole genomes could vary by geography. Recent examples include bacterial genomic analysis to inform origin of contamination of saline solution and aromatherapy with *Ralstonia pickettii* and *Burkholderia pseudomallei*, respectively [81,82].

Limitations of eDNA approaches for falsified pharmaceuticals include that it is difficult to tease out which eDNA sequences are derived from which components of the pharmaceuticals, including water used in their manufacture. Human DNA has also been described in FMs [49], raising the possibility of accruing additional actionable forensic data, as used in many other aspects of criminal forensics. Care will be needed however, to avoid inappropriate use with risks of racial profiling and to innocent bystanders (e.g., DNA of farmers who harvested plants used to produce medicine excipients) being inappropriately targeted. Also, the DNA of those leading medicine falsification activities are unlikely to be included in their ‘products’.

## 3 From evidence to intelligence: how to combine laboratory techniques and develop data analytical workflows to produce actionable forensic intelligence?

Currently, there are no established public domain FM’s laboratory workflows and data analyses frameworks specifically designed for the forensics of falsified medicines and vaccines. Returning to the questions posited here of: Q1: *Does FM (A) share its origin with another FM(B)?* Q2: *Where do the ingredients of FM(A) come from?* Q3: *Where was FM(A) produced?* and using DART, IRMS and eDNA analyses as examples, we will outline some suggestions about how techniques could be combined and what kind of data analyses could be implemented in the future to address such questions.

If a batch of unknown FM (A) samples is seized at a specific location, a first question to address is: Q1 *Does FM (A) share its origin with FM(B)?* where FM(B) could represent *reference FM samples* for which provenance information is available, or it could represent another batch of samples under investigation (e.g., collected at a different seizure) (Fig. 2). Assuming that similar bio-physico-chemical fingerprints relate to shared origin, a primary approach to answer Q1 is to conduct DART, IRMS and eDNA sequencing on whole FM products, and then perform clustering-based similarity analyses on the multivariate datasets [90,69,93]. A first set of analyses could involve the use of unsupervised algorithms (e. g., PCA, HCA) after data normalization (e.g., SNV and scaling), to reveal the structure of the data and emergent groupings [93]. A second set of analyses would involve the use of supervised algorithms (e.g., PLS-DA and SIMCA for chemometric data, Gonzalez-Dominguez et al. [93]) to categorize the samples into defined FM(A) or FM(B) groups. Supervised clustering is normally performed on a reduced number of selected features. These features represent the most differentiating signatures between the FM(A) and FM(B) groups; and are selected based on statistical comparisons of their variability distributions within- and between the groups [56] (Fig. 2).

In a best-case scenario, FM(A) would cluster together with FM (B) representing reference samples with associated geographic information. This could potentially enable answering Q3: *Where was FM(A) produced?* In a more realistic FM scenario, however, such reference samples would be missing. In such cases, clustering analyses and the process of feature selection can help selecting subsets of samples and “diagnostic” signatures in which to perform more targeted origin analyses (Fig. 2). For instance, IRMS measurements can be conducted on extracted components (e.g., excipients or water) from the selected subset of samples to address Q2: *Where do the ingredients come from?* which could provide clues to their origin. Complementary, selected discriminatory features from the eDNA datasets can be assigned to taxa to interrogate geolocation, or, if human DNA is targeted, to investigate ethnicity of possible actors involved in the FM manufacturing.

FMs’ investigations currently suffer from a lack of reference databases, containing large numbers of samples with curated laboratory data and complete seizure and circumstantial information. Such databases are critical not only for building robust classificatory models as those mentioned above, but also for investigating links between seizures, which in turn helps to understand the criminal networks behind the falsifications [54,56]. Steps should be taken in the future to create such databases, by harmonising testing and, crucially, by encouraging data sharing between pharmaceutical companies, law enforcement agencies and investigators.

## 4 Conclusions

We are still a long way from fully understanding the diversity of trade in FMs and how to locate their sources and intervene robustly. However, a variety of laboratory techniques are now available to describe in greater detail the physico-chemical and biological profiles associated with FMs. The information contained in these profiles can be used to investigate the provenance and trade links of FMs by adapting methods and data analysis frameworks that have already been developed for authenticity purposes (e.g., DART and stable isotope approaches) or that are new to the forensic field (e.g., eDNA). Future efforts should be directed at thoroughly investigating the capabilities and limitations of these techniques in the context of FM sample inter-relationships and origins, as well as building comprehensive databases that integrate FM analytical data with seizure information. Improved communication and cooperation between academic, regulatory, law enforcement agencies and the pharmaceutical industries will be crucial in countering medicine and vaccine falsification.

## Figures and Tables

**Table 1:** Laboratory approaches for investigating falsified medicines and packaging and their potential application(s) for tracing.

Investigated component	Methodological approach	Variables analysed	Format of data	Information provided/ possible tracing	References
Packaging	Visual inspection	Colour, dimensions, printing, spelling errors, holograms, barcodes and tampering	Matrix of appearance observations	-complementary information to clustering analyses	Schiavetti et al. [35] Fernandez et al. [32] Newton et al. [30]
	FTIR, VSC (Video Spectral Comparator), Raman spectroscopy, particulate analysis	Material type: composition of polymer, paper, glass, glue, ink, package and tampering	Matrix of observations and numerical values	-complementary information to clustering analyses	Lanzarotta et al. [34]
Finished product	Visual inspection	Colour, dimensions, weight, consistency, imprints (tablet, capsule)	Matrix of appearance observations	-complementary information to clustering analyses	Schiavetti et al. [35]
	Dissolution test (or dissolution profile)	Amount of API(s) released from dosage form after specific time or time points (in vitro)	Numeric value(s)	-complementary information to FM’s clustering analyses (if combined with other data)	Fernandez et al. [32]
	Colorimetry	Semi-quantification of targeted compounds (formation of colour when in contact with reagent)	Matrix of absorbance or intensity values	-complementary information to clustering analyses (if combining various colorimetric assays)	Gummadi et al. [36] Green et al. [37] Green et al. [38]
	Refractometry	(Physico-) chemical composition of (dissolved) sample based on refraction	A numeric value based on refractive index	-complementary information to clustering analyses (if combined with other data)	Green et al. [37]
	Surface morphometry and morphology, e.g., scanning electron microscopy, profilometry	2D and 3D scan of tablet surface	Matrix of numerical values associated with optical features	-complementary information to clustering analyses	Lanzarotta et al. [34]
	HPLC	Quantification of targeted compounds (mainly APIs, but other compounds also possible) based on column-separation	A numeric value (or matrix of numeric values) based on peak and retention time	-complementary information to clustering analyses	
	LC-MS	High-throughput quantification and identification of chemical compounds based on column-separation and mass-to-charge ratio detection (MS)	Matrix of numerical values associated with distinct chemical compounds present (chemical fingerprint)	-complementary information to clustering analyses	Fernandez et al. [39]
	DART-(TOF)-MS	High-throughput (semi)quantification and identification of chemical compounds based on sample ionization and mass-to-charge ratio detection of obtained analytes (MS)	Matrix of numerical values associated with distinct chemical compounds present (chemical fingerprint)	-potential use in clustering analyses for FM batch assignment	Chernetsova et al. [40] Bernier et al. [41] Gupta *et al.* [42] Fernandez et al. [39]
	IR and Raman spectroscopy	High-throughput identification of chemical compounds based on sample irradiation and measuring of absorption and emission patterns	Matrix of numerical values associated with distinct chemical compounds present (chemical fingerprint)	-potential use in clustering analyses for FM batch assignment	Ricci et al. [43] Fernandez et al. [39]
	DESI-MS	High-throughput (semi)quantification and identification of chemical compounds based on electrospray sample ionization and mass-to-charge ratio detection of obtained analytes (MS)	Matrix of numerical values associated with distinct chemical compounds present (chemical fingerprint)	-potential use in clustering analyses for FM batch assignment	Bernier et al. [41] Cardoso-Palacios et al. [44] Fernandez et al. [39]
	EDXRF	High-throughput identification of chemical compounds based on x-ray sample irradiation and measuring of diffraction patterns	Matrix of numerical values associated with distinct chemical compounds present (chemical fingerprint)	-potential use in clustering analyses for FM batch assignment	Rebiere et al. [45]
	IRMS and NMR (stable isotope analyses)	Measuring of carbon (δ^13^C), nitrogen (δ^15^N), oxygen (δ^18^O) and hydrogen (δ^2^H) isotopes in finished product or extracted excipients	A numeric value (or matrix of numeric values) based on isotope ratio composition	-potential use in clustering analyses for FM batch assignment -might inform about component origins (e.g., C4 or C3 plant origin of excipients such as starch) -could provide direct clues of geographic origin of components (e.g., comparison of H and O isotopic signature of water with isoscapes) -NMR can inform about synthetic pathways of APIs and precursors	Roncone et al. [46] Remaud et al. [47]
	eDNA analyses	High-throughput sequencing of eDNA extracted from sample	Matrix of numerical abundance values of distinct biological taxa (e. g., amplicon sequence variants)	-potential use in clustering analyses for FM batch assignment -could provide direct clues of geographic origin of product, thorough distribution ranges of assigned taxa -if human DNA, could provide ethnicity information of involved actors	Young *et al.* [48] Young et al. [49]
	Palynology and microbial culturing	-identification of biological species associated to FM	Biological species present	-complement eDNA analyses -could provide direct clues of geographic origin of product through distribution ranges of identified taxa	Wiltshire [50] Mildenhall [51]
Air in blister pack/vial/container	GC headspace sampling and GC-MS	Quantification of (gaseous) targeted compounds (e.g. residual solvent organic volatile impurities)	Matrix of numerical values associated with distinct chemical compounds present (chemical fingerprint)	-potential use in clustering analyses for FM batch assignment	Fernandez et al. [39]

## References

[1] UNDP (2024). Sustainable Development Goals.

[2] UNODC Falsified medical products (2024). Africa UWaC.

[3] WHO (2017a). A study on the public health and socioeconomic impact of substandard and falsified medical products.

[4] WHO (2017b). WHO Global Surveillance and Monitoring System for substandard and falsified medical products.

[5] WHO (2017c). Member State mechanism on substandard/spurious/falsely labelled/falsified/counterfeit (SSFFC) medical products. Appendix 3. Working definition.

[6] Ozawa S, Evans DR, Bessias S, Haynie DG, Yemeke TT, Laing SK, Herrington JE (2018). Prevalence and estimated economic burden of substandard and falsified medicines in Low- and Middle-Income Countries: a systematic review and meta-analysis. JAMA Netw Open.

[7] UNODC (2022). Trafficking in medical oroducts in the Sahel. Transnational organized Crime threat assessment — Sahel.

[8] Newton PN, Lee SJ, Goodman C (2009). Guidelines for field surveys of the quality of medicines: a proposal. PLoS Med.

[9] Saraswati K, Sichanh C, Newton PN, Caillet C (2019). Quality of medical products for diabetes management: a systematic review. BMJ Glob Health.

[10] Cavany S, Nanyonga S, Hauk C, Lim C, Tarning J, Sartorius B, Dolecek C, Caillet C, Newton PN, Cooper BS (2023). The uncertain role of substandard and falsified medicines in the emergence and spread of antimicrobial resistance. Nat Commun.

[11] Kreig M (1967). Black Market Medicine.

[12] Rooney A (2009). The Story of Medicine.

[13] Chen S, Chen Q, Askool Y, Xu L (2023). Textual research on Dioscorides and De Materia Medica. Chin Med.

[14] Newton PN, Timmermann B (2016). Fake penicillin, The Third Man`, and Operation Claptrap. BMJ.

[15] May C (2017). Transnational Crime and the Developing World.

[16] INTERPOL (2014). Pharmaceutical crime and organized criminal groups. An analysis of the involvement of organized criminal groups in pharmaceutical crime since 2008.

[17] Aljohani B, Susannah Davies AJ, Holt David (2016). Forensic Toxicology Drug use and misuse.

[18] Hall A, Koenraadt R, Antonopoulos GA (2017). Illicit pharmaceutical networks in Europe: organising the illicit medicine market in the United Kingdom and the Netherlands. Trends Organ Crime.

[19] UNODC (2010). The globalization of Crime. A transnational organized Crime threat assessment.

[20] UNODC (2011). Resolution 20/6. Countering fraudulent medicines, in particular their trafficking.

[21] Pisani E, Nistor AL, Hasnida A, Parmaksiz K, Xu J, Kok MO (2019). Identifying market risk for substandard and falsified medicines: an analytic framework based on qualitative research in China, Indonesia, Turkey and Romania. Wellcome Open Res.

[22] Yadav P, Tata HL, Babaley M (2011). The world medicines situation 2011. Storage and supply chain management.

[23] Buckley GJ, Gostin LO (2013). Countering the Problem of Falsified and Substandard Drugs.

[24] Schäfermann S, Hauk C, Wemakor E (2020). Substandard and falsified antibiotics and medicines against noncommunicable diseases in western Cameroon and northeastern Democratic Republic of Congo. Am J Trop Med Hyg.

[25] Hagen N, Hauk C, Heide L, Daniel Brombacher GM, Melanie Müller, Judith Vorrath (2022). Geopolitics of the illicit Linking the Global South and Europe.

[26] Dégardin K, Roggo Y, Margot P (2014). Understanding and fighting the medicine counterfeit market. J Pharm Biomed Anal.

[27] Kelesidis T, Falagas ME (2015). Substandard/counterfeit antimicrobial drugs. Clin Microbiol Rev.

[28] Gnegel G, Hauk C, Neci R, Mutombo G, Nyaah F, Wistuba D, Häfele-Abah C, Heide L (2020). Identification of falsified chloroquine tablets in Africa at the time of the COVID-19 pandemic. Am J Trop Med Hyg.

[29] Hauk C, Hagen N, Heide L (2021). Identification of substandard and falsified Medicines: influence of different tolerance limits and use of authenticity inquiries. Am J Trop Med Hyg.

[30] Newton PN, Fernandez FM, Plancon A (2008). A collaborative epidemiological investigation into the criminal fake artesunate trade in South East Asia. PLoS Med.

[31] Bakker-’t Hart IME, Ohana D, Venhuis BJ (2021). Current challenges in the detection and analysis of falsified medicines. J Pharm Biomed Anal.

[32] Fernandez FM, Hostetler D, Powell K, Kaur H, Green MD, Mildenhall DC, Newton PN (2011). Poor quality drugs: grand challenges in high throughput detection, countrywide sampling, and forensics in developing countries. Analyst.

[33] Vickers S, Bernier M, Zambrzycki S, Fernandez FM, Newton PN, Caillet C (2018). Field detection devices for screening the quality of medicines: a systematic review. BMJ Glob Health.

[34] Lanzarotta A, Witkowski M, Ranieri N, Albright D, Jin L, Kimani M, Elkins KM (2024). Trends in Counterfeit Drugs.

[35] Schiavetti B, Wynendaele E, Melotte V, Elst J, De Spiegeleer B, Ravinetto R (2020). A simplified checklist for the visual inspection of finished pharmaceutical products: a way to empower frontline health workers in the fight against poor-quality medicines. J Pharm Policy Pract.

[36] Gummadi S, Kommoju M (2019). Colorimetric approaches to drug analysis and applications - a review. American Journal of PharmTech Research.

[37] Green MD, Nettey H, Villalva Rojas O, Pamanivong C, Khounsaknalath L, Grande Ortiz M, Newton PN, Fernandez FM, Vongsack L, Manolin O (2007). Use of refractometry and colorimetry as field methods to rapidly assess antimalarial drug quality. J Pharm Biomed Anal.

[38] Green MD, Mount DL, Wirtz RA (2001). Authentication of artemether, artesunate and dihydroartemisinin antimalarial tablets using a simple colorimetric method. Trop Med Int Health.

[39] Fernandez FM, Green MD, Newton PN (2008). Prevalence and detection of counterfeit pharmaceuticals: a mini review. Ind Eng Chem Res.

[40] Chernetsova ES, Bochkov PO, Zatonskii GV, Abramovich RA (2011). New approach to detecting counterfeit drugs in tablets by DART mass spectrometry. Pharm Chem J.

[41] Bernier MC, Li F, Musselman B, Newton PN, Fernández FM (2016). Fingerprinting of falsified artemisinin combination therapies via direct analysis in real time coupled to a compact single quadrupole mass spectrometer. Anal Methods.

[42] Gupta S, Samal N (2022). Application of direct analysis in real-time mass spectrometry (DART-MS) in forensic science: a comprehensive review. Egypt J Forensic Sci.

[43] Ricci C, Nyadong L, Yang F, Fernandez FM, Brown CD, Newton PN, Kazarian SG (2008). Assessment of hand-held Raman instrumentation for in situ screening for potentially counterfeit artesunate antimalarial tablets by FT-Raman spectroscopy and direct ionization mass spectrometry. Anal Chim Acta.

[44] Cardoso-Palacios C, Lanekoff I (2016). Direct analysis of pharmaceutical drugs sing nano-DESI MS. J Anal Methods Chem.

[45] Rebiere H, Kermaidic A, Ghyselinck C, Brenier C (2019). Inorganic analysis of falsified medical products using X-ray fluorescence spectroscopy and chemometrics. Talanta.

[46] Roncone A, Kelly SD, Giannioti Z, Hauk C, Caillet C, Newton PN, Perez-Mon C, Bontempo L (2024). Stable isotope ratio analysis: an emerging tool to trace the origin of falsified medicines. Trends Anal Chem.

[47] Remaud GS, Bussy U, Lees M, Thomas F, Desmurs JR, Jamin E, Silvestre V, Akoka S (2013). NMR spectrometry isotopic fingerprinting: a tool for the manufacturer for tracking active pharmaceutical ingredients from starting materials to final medicines. Eur J Pharm Sci.

[48] Young JM, Linacre A (2021). Massively parallel sequencing is unlocking the potential of environmental trace evidence. Forensic Sci Int-Genet.

[49] Young JM, Liddicoat C, van Dijk KJ, Tabernero P, Caillet C, White NJ, Linacre A, Austin JJ, Newton PN (2022). Environmental DNA as an innovative technique to identify the origins of falsified antimalarial tablets-a pilot study of the pharmabiome. Sci Rep.

[50] Wiltshire PEJ, Ritz K, Dawson L, Miller D (2009). Criminal and Environmental Soil Forensics.

[51] Mildenhall DC (2017). The role of forensic palynology in sourcing the origin of falsified antimalarial pharmaceuticals. Palynology.

[52] GVR (2023). Excipients Market Size & Trends.

[53] PROGENERIKA (2020). Where do our active pharmaceutical ingredients come from? – A world map of API production.

[54] Dégardin K, Roggo Y, Margot P (2015). Forensic intelligence for medicine anti-counterfeiting. Forensic Sci Int.

[55] Dégardin K, Jamet M, Guillemain A, Mohn T (2019). Authentication of pharmaceutical vials. Talanta.

[56] Hochholdinger S, Arnoux M, Delémont O, Esseiva P (2019). Forensic intelligence on illicit markets: the example of watch counterfeiting. Forensic Sci Int.

[57] Patel NG, Rorres C, Joly DO, Brownstein JS, Boston R, Levy MZ, Smith G (2015). Quantitative methods of identifying the key nodes in the illegal wildlife trade network. Proc Natl Acad Sci USA.

[58] Ranieri N, Tabernero P, Green MD (2014). Evaluation of a new handheld instrument for the detection of counterfeit artesunate by visual fluorescence comparison. Am J Trop Med Hyg.

[59] Cody RB, Laramée JA, Nilles JM, Durst HD (2005). Direct analysis in real time (DART mass spectrometry). JEOL N.

[60] Coals P, Loveridge A, Kurian D, Williams VL, Macdonald DW, Ogden R (2021). DART mass spectrometry as a potential tool for the differentiation of captive-bred and wild lion bones. Biodivers Conserv.

[61] Price ER, McClure PJ, Huffman AN, Voin D, Espinoza EO (2022). Reliability of wood identification using DART-TOFMS and the ForeST© database: a validation study. Forensic Sci Int: Anim Environ.

[62] Sara K, Toomey V, Lorenz L (2022). Rapid-field-deployable DART-MS screening technique for 87 opioids and drugs of abuse, including fentanyl and fentanyl analogs. J Regul Sci.

[63] Bowen GJ, Revenaugh J (2003). Interpolating the isotopic composition of modern meteoric precipitation. Water Resour Res.

[64] Zachleder V, Vítová M, Hlavová M, Moudříková Š, Mojzeš P, Heumann H, Becher JR, Bišová K (2018). Stable isotope compounds - production, detection, and application. Biotechnol Adv.

[65] Ehleringer JR, Thompson AH, Podlesak DW, Bowen GJ, Chesson LA, Cerling TE, Park T, Dostie P, Schwarcz H, West JB, Bowen GJ, Dawson TE, Tu K (2010). Isoscapes: Understanding Movement, Pattern, and Process on Earth through Isotope Mapping.

[66] Kaklamanos G, Aprea E, Theodoridis G, Pico Y (2020). Chemical Analysis of Food.

[67] Perini M, Pianezze S, Strojnik L, Camin F (2019). C and H stable isotope ratio analysis using solid-phase microextraction and gas chromatography-isotope ratio mass spectrometry for vanillin authentication. J Chromatogr A.

[68] Ziegler S, Merker S, Streit B, Boner M, Jacob DE (2016). Towards understanding isotope variability in elephant ivory to establish isotopic profiling and source-area determination. Biol Conserv.

[69] Gilevska T, Gehre M, Richnow HH (2015). Multidimensional isotope analysis of carbon, hydrogen and oxygen as tool for identification of the origin of ibuprofen. J Pharm Biomed Anal.

[70] Newton PN, Chesson LA, Mayxay M, Dondorp A, Tabernero P, Howa JD, Cerling TE (2024). Forensic investigation of falsified antimalarials using Isotope Ratio Mass Spectrometry: a pilot investigation. Sci Rep.

[71] Jasper JP, Fourel F, Eaton A, Morrison J, Phillips A (2004). Stable isotopic characterization of analgesic drugs. Pharm Technol.

[72] Wang YY, Yang F, Chen J, Li YJ, Zhou J, Qing X, Yan D, Lu X, Zhou P, Zhang L (2023). Multidimensional isotope analysis of Carbon, Hydrogen, and Oxygen as a tool for traceability of lactose in drug products. J Pharm Biomed Anal.

[73] Luo X, Zhou H, Satriawan TW, Tian J, Zhao R, Keenan TF, Griffith DM, Sitch S, Smith NG, Still CJ (2024). Mapping the global distribution of C(4) vegetation using observations and optimality theory. Nat Commun.

[74] Armellin S, Brenna E, Fronza G, Fuganti CJ, Pinciroli M, Serra S (2004). Establishing the synthetic origin of amphetamines by ^2^H NMR spectroscopy. Analyst.

[75] Carter JF, Murray M, Sleeman R (2002). Isotopic characterisation of 3,4-methylenedioxyamphetamine and 3,4-methylenedioxymethylamphetamine (ecstasy). Analyst.

[76] Acetti D, Brenna E, Fronza G, Fuganti C (2008). Monitoring the synthetic procedures of commercial drugs by ^2^H NMR spectroscopy: the case of ibuprofen and naproxen. Talanta.

[77] Bussy U, Thibaudeau C, Thomas F, Desmurs JR, Jamin E, Remaud GS, Silvestre V, Akoka S (2011). Isotopic finger-printing of active pharmaceutical ingredients by ^13^C NMR and polarization transfer techniques as a tool to fight against counterfeiting. Talanta.

[78] Byrd J, Sutton L (2020). Forensic entomology for the investigator. Wiley Interdiscip Rev: Forensic Sci.

[79] Yuan HY, Wang ZW, Wang Z, Zhang FY, Guan DW, Zhao R (2023). Trends in forensic microbiology: from classical methods to deep learning. Front Microbiol.

[80] Haarkotter C, Saiz M, Galvez X, Medina-Lozano MI, Alvarez JC, Lorente JA (2021). Usefulness of microbiome for forensic geolocation: a review. Life (Basel).

[81] Chen YY, Huang WT, Chen CP, Sun SM, Kuo FM, Chan YJ, Kuo SC, Wang FD (2017). An outbreak of Ralstonia pickettii bloodstream infection associated with an intrinsically contaminated normal saline solution. Infect Control Hosp Epidemiol.

[82] Gee JE, Bower WA, Kunkel A (2022). Multistate outbreak of Melioidosis associated with imported aromatherapy spray. N Engl J Med.

[83] Keim PS, Budowle B, Ravel J, Budowle B, Breeze RG, Morse SA, Schutzer SE, Keim P (2011). Microbial Forensics.

[84] Griffin A, Kirkbride KP, Henry J, Painter B, Linacre A (2021). DNA on drugs! A preliminary investigation of DNA deposition during the handling of illicit drug capsules. Forensic Sci Int-Genet.

[85] Taberlet P, Coissac E, Hajibabaei M, Rieseberg LH (2012). Environmental DNA. Mol Ecol.

[86] Satam H, Joshi K, Mangrolia U (2023). Next-generation sequencing technology: current trends and advancements. Biol-Basel.

[87] Compson ZG, McClenaghan B, Singer GAC, Fahner NA, Hajibabaei M (2020). Metabarcoding from Microbes to mammals: comprehensive bioassessment on a global scale. Front Ecol Evol.

[88] Antil S, Abraham JS, Sripoorna S (2023). DNA barcoding, an effective tool for species identification: a review. Mol Biol Rep.

[89] Coghlan ML, Haile J, Houston J, Murray DC, White NE, Moolhuijzen P, Bellgard MI, Bunce M (2012). Deep sequencing of plant and animal DNA contained within traditional Chinese medicines reveals legality issues and health safety concerns. PLoS Genet.

[90] Foster NR, Taylor D, Hoogewerff J, Aberle MG, de Caritat P, Roffey P, Edwards R, Malik A, Waycott M, Young JM (2023). The secret hidden in dust: assessing the potential to use biological and chemical properties of the airborne fraction of soil for provenance assignment and forensic casework. Forensic Sci Int-Genet.

[91] Wirta H, Abrego N, Miller K, Roslin T, Vesterinen E (2021). DNA traces the origin of honey by identifying plants, bacteria and fungi. Sci Rep.

[92] Khansaritoreh E, Salmaki Y, Ramezani E, Azirani TA, Keller A, Neumann K, Alizadeh K, Zarre S, Beckh G, Behling H (2020). Employing DNA metabarcoding to determine the geographical origin of honey. Heliyon.

[93] Gonzalez-Dominguez R, Sayago A (2022). Fernandez-Recamales A, An overview on the application of chemometrics tools in food authenticity and traceability. Foods.

